# Thickness-Dependent Crystallization of Ultrathin Antimony Thin Films for Monatomic Multilevel Reflectance and Phase Change Memory Designs

**DOI:** 10.1021/acsami.1c23974

**Published:** 2022-03-10

**Authors:** Daniel T. Yimam, Bart J. Kooi

**Affiliations:** Zernike Institute for Advanced Materials, University of Groningen, Nijenborgh 4, 9747 AG Groningen, The Netherlands

**Keywords:** monatomic phase change materials, antimony, thickness-dependent crystallization, dynamic ellipsometry, pulsed laser deposition, multilevel reflectance, nanophotonics

## Abstract

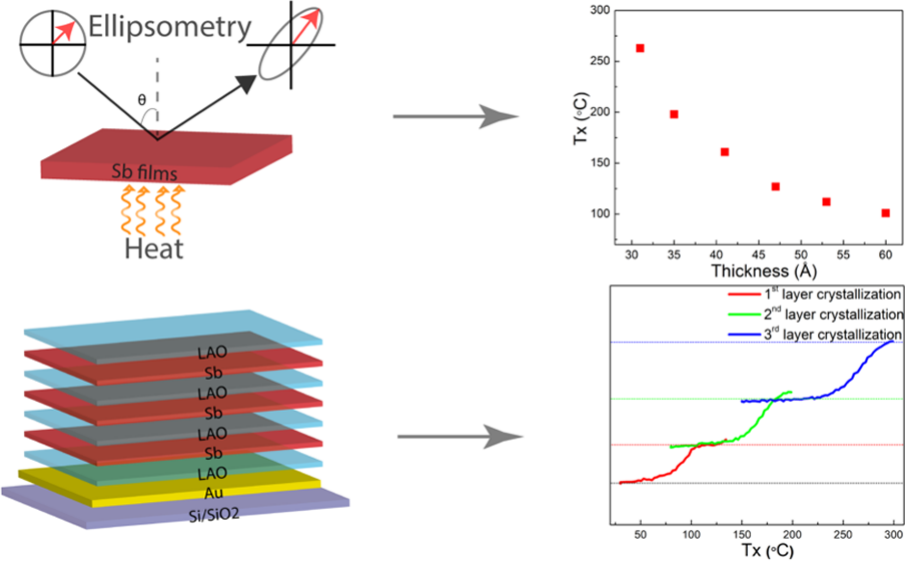

Phase change materials, with more than one reflectance and resistance states, have been a subject of interest in the fields of phase change memories and nanophotonics. Although most current research focuses on rather complex phase change alloys, *e.g.*, Ge2Sb2Te5, recently, monatomic antimony thin films have aroused a lot of interest. One prominent attractive feature is its simplicity, giving fewer reliability issues like segregation and phase separation. However, phase transformation and crystallization properties of ultrathin Sb thin films must be understood to fully incorporate them into future memory and nanophotonics devices. Here, we studied the thickness-dependent crystallization behavior of pulsed laser-deposited ultrathin Sb thin films by employing dynamic ellipsometry. We show that the crystallization temperature and phase transformation speed of as-deposited amorphous Sb thin films are thickness-dependent and can be precisely tuned by controlling the film thickness. Thus, crystallization temperature tuning by thickness can be applied to future memory and nanophotonic devices. As a proof of principle, we designed a heterostructure device with three Sb layers of varying thicknesses with distinct crystallization temperatures. Measurements and simulation results show that it is possible to address these layers individually and produce distinct and multiple reflectance profiles in a single device. In addition, we show that the immiscible nature of Sb and GaSb could open up possible heterostructure device designs with high stability after melt-quench and increased crystallization temperature. Our results demonstrate that the thickness-dependent phase transformation and crystallization dynamics of ultrathin Sb thin films have attractive features for future memory and nanophotonic devices.

## Introduction

Since the introduction of the concept of monatomic phase change memory,^1^ interest in the behavior of ultrathin Sb films has increased substantially. Phase change materials (PCMs) are, in general, rather complex alloys because they have to satisfy a range of requirements, which then can be engineered by fine-tuning ternary or even quaternary alloys. Although such alloys can have desired properties in their initial states, they also come with challenges because when switched in nanoscale volumes repeatedly between the crystalline and amorphous phase *via* the liquid phase and when subjected to high temperatures and electrical fields (gradients), they can also decompose and then lose their favorable properties.^2−4^ A PCM based on a single element would of course not suffer from such challenges. However, for a single element it is typically impossible to combine amorphous phase stabilities at operating temperatures (*e.g.*, up to 100 °C) for good data retention with high crystallization speeds (*e.g.*, at 500 °C) required for fast switching of the memory. Selenium is the only monatomic material that is a good glass former,^5^ but it does not provide the desired property contrast and fast crystallization kinetics required for memory applications.

Antimony offers property contrast and fast crystallization kinetics (even explosive crystallization^6,7^), but it does not have the amorphous phase stability for proper data retention. Now, in the seminal works on monatomic phase change memory, it was demonstrated that when Sb is confined in ultrathin films (sandwiched between oxides), the amorphous phase stability and thus data retention could be strongly improved.^1,8^ In some later works, it was shown that devices with such ultrathin Sb films could be exploited in (1) neuromorphic computing (due to low resistance drift)^9^ and (2) plasmonics-enabled nanophotonic and optoelectronic applications.^10^ In the former work, a crystallization temperature (*T_x_*) of 154 °C (with a heating rate of 40 °C min^–1^) was observed for a 4 nm Sb film and in the latter work, a *T_x_* of 127 °C (with a heating rate of ∼150 °C min^–1^) was observed for a 5 nm Sb film. However, as far as the authors know, no work has shown any precise dependence of *T_x_* on Sb film thickness, and this will be demonstrated in the present work for both capped and uncapped Sb films. Moreover, this precise dependence can be exploited to make a multilayer structure where different Sb films can be crystallized and addressed individually because they require different temperatures and thus Joule heating pulses for their switching. This can, for instance, be used to design multilevel reflectance states as shown in the present work with very good agreement between experimental and simulated results. Finally, we demonstrate that ultrathin Sb films can also play a role in PCM heterostructure designs to tailor the crystallization of the adjacent PCM, even without the risk of any intermixing at the interface.

## Results and Discussion

A series of ultrathin amorphous Sb films with uniform film thicknesses in the range between 3.1 and 6.0 nm were deposited at room temperature using pulsed laser deposition (PLD). This series was obtained by varying the number of laser pulses between 270 and 500 and keeping other deposition parameters constant. Spectroscopic ellipsometry (SE) was used to determine the relation between the number of pulses and the film thicknesses; see Figure S1a and accompanying text providing details of the procedure to assess film thickness using SE. The film thicknesses derived in this way can be considered accurate because a very similar film thickness, 6.3 nm, was obtained using AFM analysis for the 6.0 nm thick film derived from SE analysis; see Figure S1b,c. The films have a very uniform thickness, as can be derived from AFM images and cross-sectional transmission electron microscopy (TEM) images. Examples of these images are shown in Figures S1 and S2, respectively. The amorphous nature of as-deposited films was confirmed using high-resolution transmission electron microscopy and selected-area electron diffraction, where the example results are shown in Figure S2a,b, respectively.

**Figure 1:**
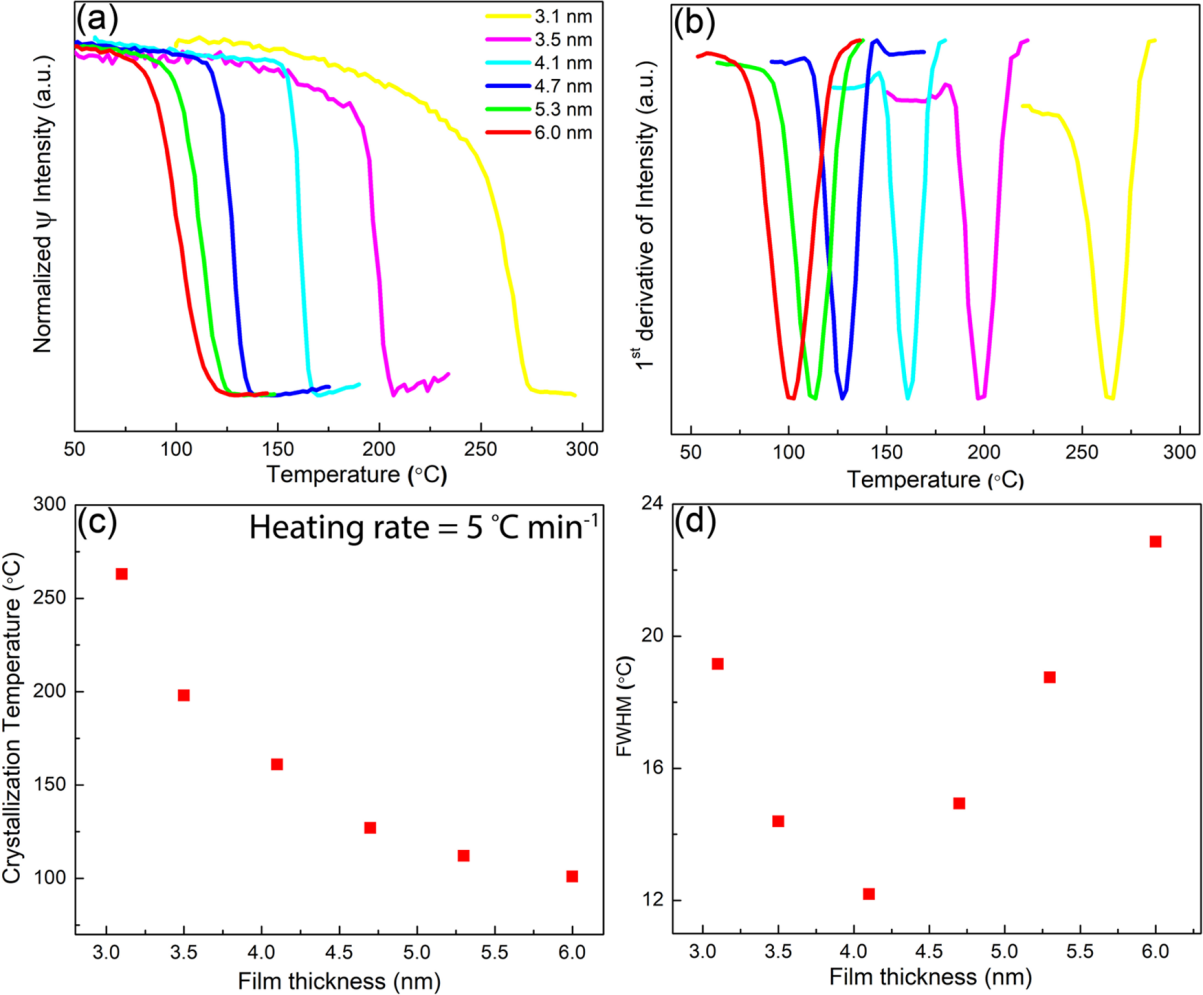
(a) Intensity change due to phase transformation from dynamic ellipsometry measurement variable ψ for variable thicknesses of Sb thin films. (b) First derivative of the measured intensity for crystallization temperature determination. (c) Thickness *vs* crystallization temperature data extracted from dynamic ellipsometry measurement. All samples were uncapped and a heating rate of 5 °C min^–1^ was used for heating. (d) The FWHM *vs* film thickness shows that 4 nm Sb thin films crystallize faster. The FWHM values were extracted from panel (b).

Ellipsometry is a particularly sensitive method to monitor the amorphous-to-crystalline phase change in these ultrathin Sb films. Figure 1a shows the results of dynamic ellipsometry (DE), where a heating rate of 5 °C min^–1^ was employed for the series of Sb films. The normalized intensity along the vertical axis is based on the measured ψ parameter during heating; see Sections S1 and S3. Figure 1a shows a very pronounced drop in intensity upon crystallization of the films. Moreover, the thinner the film, the higher the temperature where this drop occurs. To accurately determine the crystallization temperature, the first derivatives of the intensity versus temperature curves are taken as depicted in Figure 1b, and then the peak in these derivative curves, corresponding to the highest crystallization rate, is taken as a measure of the crystallization temperature, *T_x_*. Figure 1c demonstrates that *T_x_* strongly depends on the Sb film thickness, where for a thickness of 6.0 nm (500 pulses), *T_x_* is about 100 °C, but it increases to about 260 °C for a film thickness of 3.1 nm (270 pulses).

The results shown in Figure 1 hold for uncapped Sb films and the ellipsometry analysis was performed within 48 h after the samples were deposited and taken out of the vacuum chamber. Then, of course one can question if the results in Figure 1 are affected by air exposure of the ultrathin Sb films. To verify this, also a series of LaAlO_3_-capped Sb films were produced. This (4–5 nm thick) LAO capping was deposited onto the Sb films without breaking the vacuum. The DE analysis of these capped films depicted in Figure S3 shows that the results in Figure 1 are very well reproduced. In the case of the 500 pulses film, *T_x_* = 106 and 101 °C for the capped and uncapped films, respectively. In the case of the 300 pulses film, *T_x_* = 192 and 198 °C for the capped and uncapped films, respectively. Only in the case of the 400 pulses film, there is a clear difference, 149 and 127 °C for the capped and uncapped film, respectively. Interestingly, the capped films show that the 250 and 200 pulses films could not be crystallized at a temperature up to 300 °C and thus have *T_x_* higher than 300 °C. This is in line with the uncapped film, where the 270 pulses (3.1 nm thick) film already shows a *T_x_* of 263 °C and, therefore, *T_x_* must be higher for thinner films produced with less pulses. Although the 250 and 200 pulses films do not show crystallization, still at temperatures beyond about 200 °C, there is lowering in the ellipsometry intensity that can probably be associated with a structural relaxation of the amorphous phase (toward a more stable structure). In contrast, the (capped) 600 pulses film (about 7.2 nm thick) does not show any change in ellipsometry intensity because it is already crystalline at the start. Also, a 1000 pulses uncapped film was already crystalline at the start (data not shown).

The ellipsometry data in Figure 1a not only provide a measure of *T_x_* but also contain information about the overall crystallization rate from how fast the intensity drops upon crystallization and thus from the width of the peak (*e.g.*, FWHM) in Figure 1b. The FWHM values, extracted from a Gaussian fit of the first derivative curves in Figure 1b, are depicted in Figure 1d. These results show that the crystallization rate increases and thus the FWHM decreases, when the Sb film thickness reduces from 6.0 down to 4.1 nm, but then the crystallization rate decreases when going to thinner films. Apparently, here, two competing effects are at play. For thinner films, the crystallization temperature increases and at a higher temperature, there is more atomic mobility and the crystallization rate can therefore increase. However, there is also the effect of increased confinement for thinner films that retards the crystal growth.^11^ Note that Sb films show an extremely growth-dominated crystallization behavior. Earlier results on 200 nm thick Sb films (containing 7 atom % Ge) show crystal sizes in the order of millimeters.^12^ Crystal sizes increase when lowering the Ge concentration from 8 to 6 atom %. Apparently, the optimum overall crystallization rate for the ultrathin pure Sb films occurs here for a film thickness at 4.1 nm and a *T_x_* of about 160 °C. For thicker films and thus lower *T_x_*, the crystallization rate is limited by insufficient atomic mobility due to low temperatures and for thinner films and higher *T_x_*, the crystallization rate is limited by insufficient mobility due to confinement. Indeed, it is a well-known effect that by confinement, the effective atomic mobility and crystal growth rate are reduced due to an increase in viscosity that can be associated with an increase in the glass transition temperature.^13−18^ Apparently, for our thinnest Sb films, the effect of confinement is so strong that despite the increasing temperature at which crystallization occurs, the crystallization rate nevertheless decreases.

**Figure 2:**
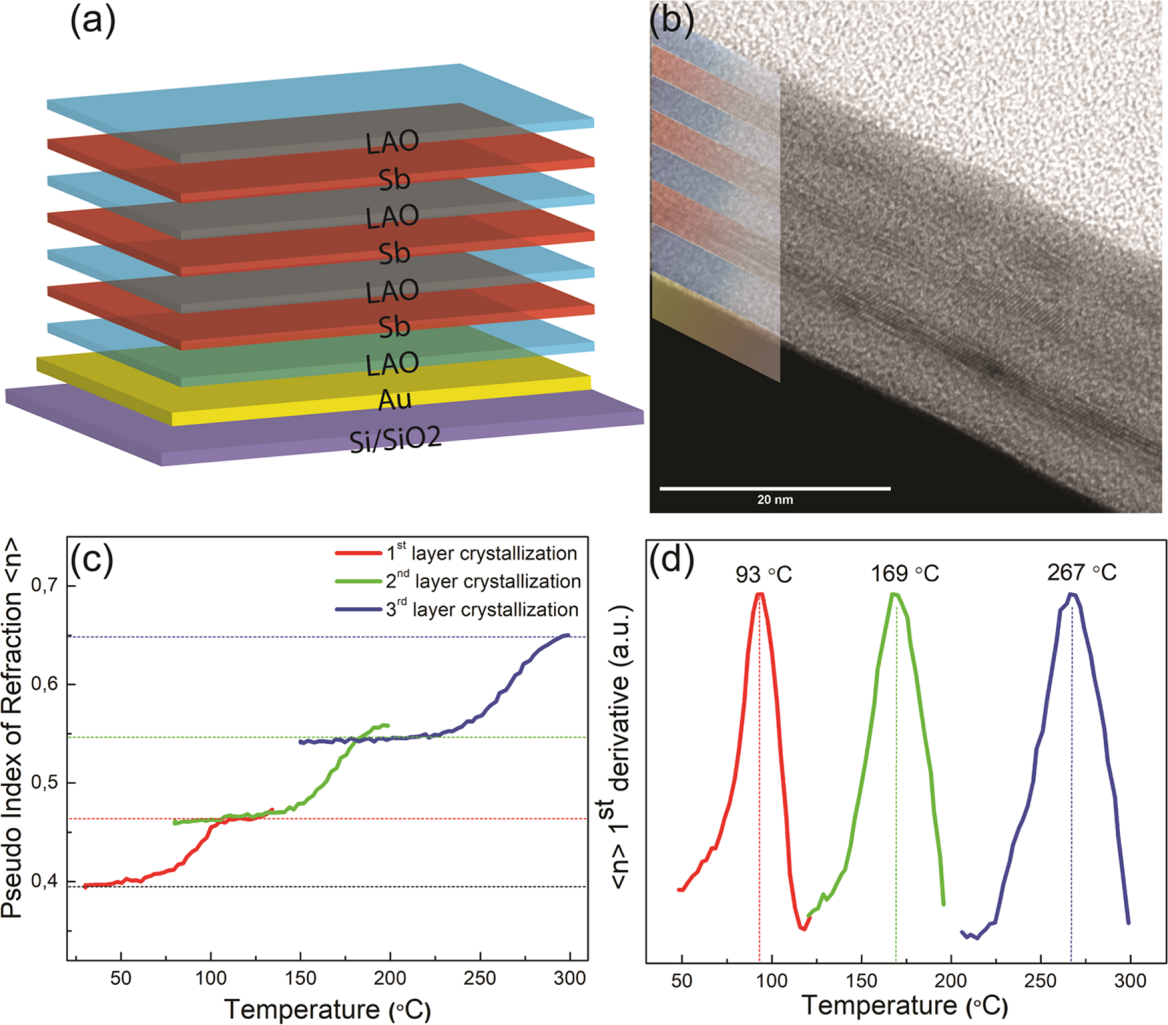
(a) Schematic of a heterostructure design for the monatomic multilevel reflectance device. Three layers of Sb thin films with variable thickness, separated by transparent LaAlO_3_ (LAO) layers, are deposited on a gold substrate. (b) Cross-sectional TEM image of the heterostructure device showing individual layers corresponding to the schematic. (c) Pseudo-index of refraction ⟨*n*⟩ from dynamic ellipsometry measurement indicating individual crystallizations of the Sb layers in the heterostructure. (d) First derivative of ⟨*n*⟩ provides the crystallization temperature for individual layer phase transformation.

The strong dependence of *T_x_* on the film thickness can be exploited in data storage and optoelectronic applications, particularly for creating multilevel states. Usually, multilevel reflectance states are produced by partial crystallization (or amorphization) in localized regions of a sample. This partial switching is generally achieved with a pump–probe setup with a different and discrete laser energy.^19,20^ The structural transformation relative to the laser power will produce multiple reflectance states. Another approach to multilevel reflectance spectra is the possibility of individual phase switching in an optical heterostructure device consisting of multiple phase change material alloys like Sb_2_Te_3_, GeTe, and GST.^21,22^ Although the results produced can be considered promising, the downside is that the individual layers in the heterostructure optical device each have a fixed *T_x_*, and the tuning reflectance in a specific temperature range can be problematic. Sb thin films provide a solution for this since the *T_x_* for a layer can be directly controlled by the film thickness. In addition, compared to known PCM alloys, the “monatomic” aspect of Sb thin films also resolves multiple problems related with device production and endurance. As a proof of principle, we here constructed a single device where on a Si wafer covered with thermal oxide, first, a (100 nm thick) Au bottom layer (reflector) was deposited and then three Sb layers sandwiched between LAO layers, as schematically depicted in Figure 2a; see also Figure S2c. The bottom Sb layer is the thickest one as produced with 500 pulses, the middle layer with 300 pulses, and the top layer is the thinnest one as produced with 270 pulses.

A cross-sectional TEM image of the device is shown in Figure 2b, clearly highlighting the three Sb films sandwiched between amorphous oxide layers. The initially amorphous Sb layers were completely crystallized during dynamic ellipsometry, as can be observed in the TEM image. The dark contrast produced by the three Sb layers reduces from bottom to top, reflecting the reduced Sb film thickness from bottom to top. During the heating and cooling cycles, the three different Sb films are crystallized individually, where heating to about 135 °C is sufficient to fully crystallize the thickest (500 pulses) layer, having a *T_x_* of 93 °C, but still keeps the other two thinner Sb films amorphous; see Figure 2d. Then, reheating to about 200 °C fully crystallizes the intermediate thickness (300 pulses) layer, having a *T_x_* of 169 °C, but still keeps the thinnest Sb film amorphous. Finally, when reheating to about 300 °C, the thinnest (270 pulses) layer, having a *T_x_* of 267 °C, is fully crystallized. This process of overall heating demonstrates that it should be possible to also use Joule heating *via* laser or electrical pulses with different powers to individually crystallize such Sb films with distinct crystallization temperatures. Of course, such a multilayer structure only becomes interesting when it can produce an additional performance like multilevel optical or electrical states. The results in Figure 2c indeed show that distinct optical states are produced; in this case, the pseudo index of refraction ⟨*n*⟩ of the overall structure for a wavelength of 900 nm (as explained in more detail in Section S3). When all three Sb layers are amorphous, ⟨*n*⟩ is about 0.40. When only the bottom Sb layer is crystallized, ⟨*n*⟩ is about 0.47. Crystallizing the intermediate layer leads to an ⟨*n*⟩ of about 0.55 and, finally, when all three layers are crystallized, ⟨*n*⟩ becomes 0.65. Hence, distinct property contrast is generated between four states.

**Figure 3:**
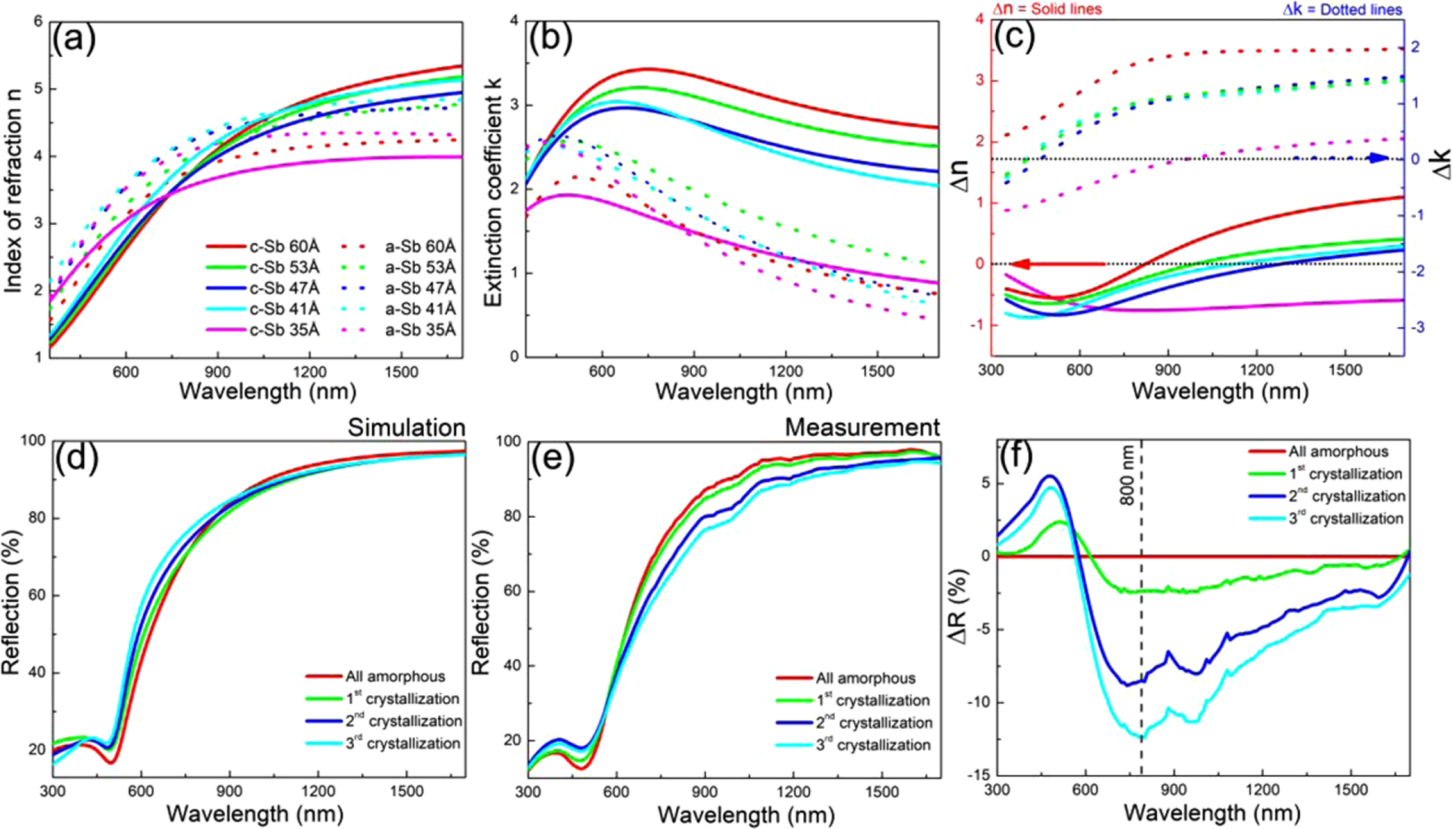
(a, b) Index of refraction *n* and extinction coefficient *k* extracted from spectroscopic ellipsometry measurement for both as-deposited (a-Sb) and annealed (c-Sb) Sb thin films with variable thicknesses. (c) Change in the index of refraction (Δ*n*) and extinction coefficient (Δ*k*) induced by phase transformation from as-deposited to annealed. (d) Simulated and (e) measured reflectance spectra for the heterostructure design (shown in Figure 2a,b) consisting of three Sb layers with varying thicknesses. Individual layer crystallizations produced distinct reflectance profiles. (f) Change in reflectance spectral values due to individual layer transformations. The maximum reflectance change is shown at 800 nm.

Fitting a model to the raw data, spectroscopic ellipsometry allows extraction of optical properties of the (uncapped) Sb films (analyzed in Figure 1) like the index of refraction *n* and extinction coefficient *k* for a spectral range. In this case, the wavelength range is from 300 to 1700 nm. All Sb layers are analyzed first in the amorphous state and then after crystallization. The results are shown in Figure 3a–c. Some general trends can be discerned, where the thinnest (3.5 nm) crystalline film is somewhat an outlier, but more on this below. The *n* and *k* spectra do not reflect that all films consist of identical antimony but tend to be film thickness-dependent. The *n* of the amorphous films tends to be higher than the *n* of the crystalline films for the lower wavelength range typically between 300 and 900 nm, but this reverses for the higher wavelength range (from 900 to 1700 nm). The *k* of the amorphous films is significantly lower than the *k* of the crystalline films, at least for the wavelength range between 600 and 1700 nm but vanishes for wavelengths below 600 nm. Compared to the other crystalline films, the *n* and *k* of the thinnest crystalline film show a rather deviating behavior. Interestingly, the *n* and *k* of the thinnest crystalline film are remarkably similar to the ones of the thickest amorphous film. The *n* and *k* spectra of our antimony films have some similarities and differences with previously reported *n* and *k* values of sputtered antimony films.^10^ Starting from the similarities and being thickness-dependent, both results present somewhat comparable values of *n* and *k* for 3–6 nm thick films. Moreover, both works report an outlier result for the thinner crystalline antimony thin films with lower values of optical constants, where below, we will give an explicit explanation for this outlying behavior. In addition, in both results, the change in extinction coefficient (Δ*k*) upon crystallization is relatively bigger than the refractive index change (Δ*n*). However, some differences are also noted. The most obvious one is the stability of the as-deposited amorphous phase in the sputtered antimony thin films. A substantial optical contrast for film thicknesses up to 15 nm has been reported when crystallizing the (as-deposited) sputtered antimony films. Here, the optical contrast for pulsed laser-deposited Sb films can only be sustained for film thicknesses up to 6.0 nm because thicker films are already deposited in the crystalline form and cannot be switched anymore. This indicates that the pulsed laser-deposited films have a structure closer to the equilibrium structure, *i.e.*, directly crystalline for films thicker than 6.0 nm and with a structure probably closer to the melt-quenched one for thinner films. It is a well-known effect that melt-quenched amorphous PCMs are often less thermally stable than the same PCMs in as-sputtered films.^23^

Although it may seem rather speculative at this moment, follow-up work (with detailed atomic structure analysis) will show that this special behavior of the thinnest crystalline film can be associated with the chemical bonding that prevails between the Sb atoms. In the amorphous films, the bonding is covalent. In crystalline Sb, the bonding is metallic-like, but this is a special type of metallic bonding that has been coined metavalent bonding (MVB).^13^ The bonding in Sb occurs solely by p electrons and, on average, three p electrons are available per Sb atom. Although rhombohedrally distorted, the structure of Sb is close to simple cubic,^13^ implying that the coordination is close to 6-fold. For covalent bonding, six electrons are needed per Sb atom. However, only three electrons are available. This means a half-filled band and thus, to make stable bonds, the electrons delocalize and the bonding becomes metallic-like. However, for such a half-filled band, the structure can lower its energy by Peierls distortion, where each Sb atom makes three strong (more covalent like) bonds and three weaker (more van-der-Waals like) bonds. Indeed, in crystalline Sb, such Peierls distortion occurs, leading to the rhombohedral distortion of the simple cubic structure and to dimerization, *i.e.*, bilayer formation.^24^ Our current ongoing work on the growth and film structure shows that thin crystalline Sb films tend to be highly textured, where all domains have their *c*-axis out of plane. Now, what could be the reason that the thinnest crystalline Sb films clearly deviate from the thicker crystalline films and are close to the amorphous ones? Evidence is increasing that the MVB is breaking down for ultrathin films.^13^ The bonding in these strongly confined crystalline films again becomes more covalent. A likely mechanism is that the Peirels distortion increases in the ultrathin films and this is opening a band gap. So, there is a transition from electron delocalization (by MVB) in thicker crystalline films to electron localization in ultraconfined crystalline films. (Electrons are anyhow localized in the amorphous films.) This could well explain, to a large extent, the observed behavior in Figure 3a–c.

Now, when knowing the *n* and *k* spectra for the individual Sb films in both amorphous and crystalline states from Figure 3a–c and also knowing the *n* and *k* spectra for LAO (spacer and capping layers) and for Au (reflector layer), it is possible to simulate the behavior of the structure in Figure 2a,b. The reflection (15° off-normal) has been simulated in Figure 3d for all three amorphous Sb films and gradual crystallization of the thicker to thinner Sb films till all three Sb films are crystalline. This generates four reflection spectra. As a follow-up of the results in Figure 2, the same four spectra can of course also be measured experimentally as shown in Figure 3e. The results in Figure 3d,e agree to a large extent, providing confidence about the reliability of the *n* and *k* spectra derived in Figure 3a,b for the individual layers. In both measured and simulated spectra, it can be seen that there is a crossover: for long wavelengths (900–1700 nm), there is a high reflectivity that decreases with increasing number of crystallized films, but for short wavelengths (300–600 nm), there is a low reflectivity that increases with increasing number of crystallized films. In general, these films thus reflect strongly in the IR and weakly in the visible range, but this contrast decreases somewhat upon gradual crystallization of the different Sb layers. In Figure 3f, the relative changes in reflectivity for the experimental spectra are provided with respect to the initial state where all three Sb films are amorphous. Here, it is particularly emphasized that for wavelengths longer than 600 nm, the reflectivity decreases upon sequential crystallization of the Sb layers and that the biggest relative change occurs for a wavelength of about 800 nm. To a lesser extent, the opposite effect occurs for wavelengths shorter than 600 nm.

**Figure 4:**
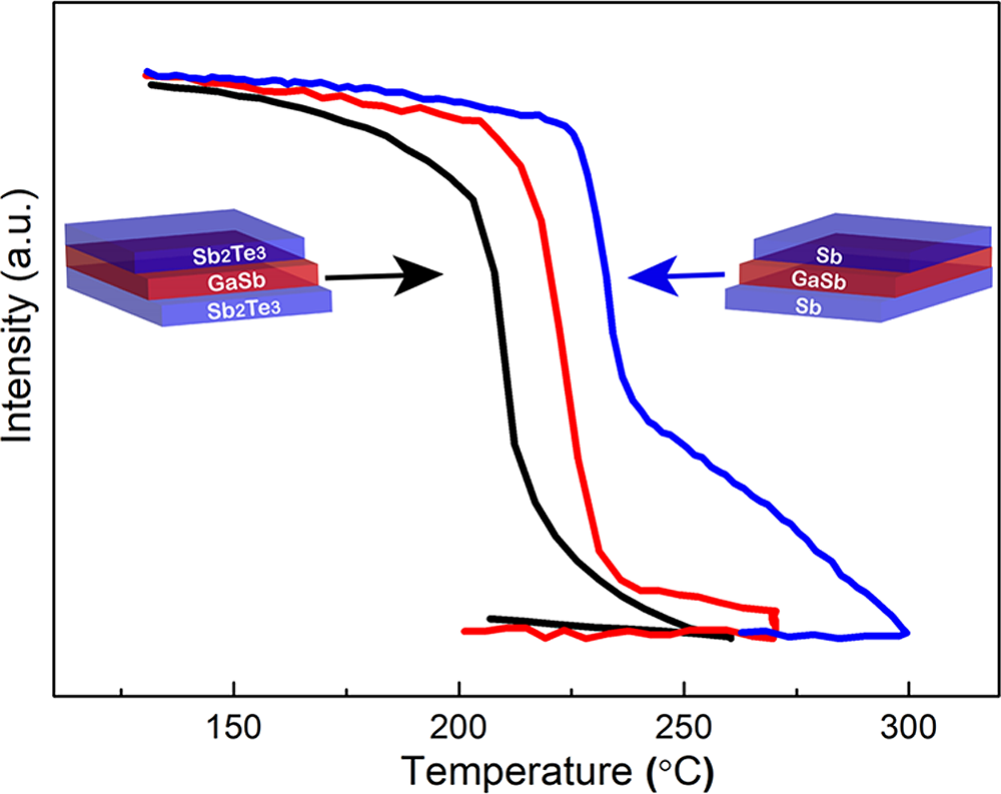
Dynamic ellipsometry measurement results show phase transformation of a monolithic stoichiometric GaSb thin film (red), a GaSb layer sandwiched between thin Sb_2_Te_3_ layers (black), and a GaSb layer sandwiched between ultrathin Sb layers (blue). Sb_2_Te_3_ layers reduce the crystallization temperature, while the Sb layers increase it.

The present work demonstrates that PLD allows accurate control of Sb film thickness and that by tuning the thickness, the amorphous phase stability and thus the crystallization temperature can be controlled well. This control is highly useful for memory and optoelectronic applications and can also be exploited very well in heterostructure designs as demonstrated here. Using PLD, we produced heterostructures based on a central GaSb film sandwiched between either Sb_2_Te_3_ or ultrathin Sb layers with all of the materials deposited at room temperature. Note that the GaSb film has a stoichiometric composition, which we realized recently,^25^ in contrast to most previous work where, for instance, a composition of Ga_45_Sb_55_ was achieved.^26^Figure 4 shows the results of dynamic ellipsometry for both heterostructures in direct comparison with the monolithic GaSb (*i.e.*, when Sb_2_Te_3_ or ultrathin Sb sandwich layers would be absent). The monolithic GaSb shows a *T_x_* of about 225 °C. When sandwiched between Sb_2_Te_3_, the *T_x_* reduced to about 200 °C. On the other hand, when sandwiched between ultrathin (250 pulses) Sb, the *T_x_* increases to about 250 °C and even gradual further crystallization occurs till 300 °C. So, these heterostructure results demonstrate that Sb_2_Te_3_ facilitates crystallization of GaSb and ultrathin Sb retards it.

Two important phenomena are at play here: (1) the *T_x_* of Sb_2_Te_3_ is lower than the one of GaSb and therefore Sb_2_Te_3_ is already crystalline and acts as a template for GaSb crystallization and thus accelerates GaSb crystallization. Indeed, this behavior of Sb_2_Te_3_ as a template accelerating crystallization and reducing the crystallization temperature is known from previous work on GeTe thin films.^27^ On the other hand, the 250 pulses Sb film has by itself a *T_x_* close to 300 °C. When GaSb wants to crystallize, the adjacent Sb is still amorphous and retards this crystallization. When GaSb is crystallized at 250 °C, it acts as a template for Sb crystallization and although the Sb is ultrathin, its crystallization only proceeds very gradually in the 250–300 °C range. (2) Sb_2_Te_3_ and GaSb have a tendency to react and intermix at the interface (forming the GaSbTe alloy from the Sb_2_Te_3_ and GaSb tie-line),^28,29^ whereas Sb and GaSb do not react and are immiscible at their interface. Also, this difference could play an important role in either accelerating or retarding crystallization at the interface. Moreover, such a heterostructure based on immiscible phases should remain very stable during operation, *e.g.*, during repeated melt quenching because any liquid composition in the range between stoichiometric GaSb and Sb will phase separate back into nearly stoichiometric GaSb and pure Sb (and then will be guided back *via* the multilayer structure surrounding the molten volume).

One can question whether the rather strong dependence of *T_x_* on film thickness observed here is specific for Sb films or a general feature of PCM films. Indeed, for various PCM films, also comprising the prototypical material Ge_2_Sb_2_Te_5_, strong thickness dependences have been observed.^13^ Still, our current ongoing work on alloying Sb with Ge shows that the strong thickness dependence is largely reduced in this case. Of course, by alloying Sb with *e.g.*, 5–10 atom %, the amorphous phase stability is largely increased and thereby the *T_x_* for films thicker than 10 nm is 100–200 °C higher than for pure Sb.^12^ When reducing the film thickness toward 3 nm, again an increase in *T_x_* is observed, but this increase is not as pronounced as for pure Sb. For a 3 nm film, *T_x_* ends up similarly as the one for pure Sb and even slightly lower.

Another important issue is whether the present results are sensitive toward the type of substrate material other than SiO_2_ used here. We show that capping with another oxide (LAO) has no significant effect. However, it is very likely that when the amorphous (oxide or nitride) substrate is, for instance, replaced by a crystalline metal, the results will change significantly and this may even include reversing the effect, *i.e.*, instead of stabilizing the amorphous phase by confinement in ultrathin films, the amorphous phase gets less stable by such a confinement.^13^ More future work on pure Sb films is required to clarify this substrate dependence.

The Sb films analyzed here are as-deposited. It is well known that the behavior of the material can change significantly when going from as-deposited to melt-quenched. For some alloys, this effect can be very dramatic, but there are also alloys where the effect is rather limited.^23^ A dramatic one is for instance sputter-deposited Ga_8_Sb_77_Te_15_ where *T_x_* drops from 230 to 84 °C when switching from as-deposited to melt-quenched, where the latter step was achieved using laser pulses. On the other hand, for Ge_15_Sb_85_ and Ga_15_Sb_85_ alloys, the effect is not so bad because *T_x_* drops from 250 to 208 °C and from 233 to 210 °C, respectively. In our case, films are not sputter deposited, but grown using PLD. There are some indications that PLD generates structures more close to the melt-quenched ones than (magnetron) sputtering. For instance, when we optimize deposition of Sb_2_Te_3_ films at room temperature with the aim to produce fully amorphous films, we did not succeed because we ended up with an amorphous matrix containing nanoscale crystalline seeds. On the other hand, with (magnetron) sputtering at RT, it is rather easy to achieve fully amorphous Sb_2_Te_3_ films. Therefore, we expect that our current results for as-deposited ultrathin Sb films will not change dramatically when producing melt-quenched structures in the films. Still, checking this carefully should be the aim of the future research.

## Conclusions

Ultrathin Sb films with uniform coverage and precisely tuned film thickness can be produced using pulsed laser deposition. Room-temperature-deposited films with a thickness less than 7 nm have an amorphous structure and thicker films have a crystalline structure when deposited on amorphous silicon oxide or nitride substrates. Crystallization of these ultrathin films can be monitored sensitively using dynamic ellipsometry. The crystallization temperature (*T_x_*) is strongly temperature-dependent where *T_x_* can be tuned for instance between 100 and 260 °C when varying the film thickness between 6 and 3 nm, respectively. The crystallization speed shows a maximum for a film thickness of 4 nm. Spectroscopic ellipsometry (SE) shows that, particularly, the extinction coefficient markedly increases upon crystallization in the wavelength range of 600–1700 nm. Interestingly, there is also a clear transition between the 3.5 and 4.1 nm crystalline Sb films, where the properties of the 3.5 nm crystalline film still resemble the ones of the amorphous films. It is very likely that this is a transition from metallic-like bonding in the thicker crystalline films to covalent bonding (with localized electrons) in the thinner crystalline films, which then stays similar to the bonding in the amorphous films.

The dependence of *T_x_* on Sb film thickness can be exploited in memory and nanophotonics devices. As a proof of principle, we constructed a multilayer device where three Sb films with different thicknesses are sandwiched between thin oxide spacer layers. The Sb layers can be switched individually and provide distinct optical states, as demonstrated experimentally and reproduced well by simulation. Also, in heterostructures, the ultrathin Sb films can be exploited, where we show that the *T_x_* of stoichiometric GaSb was increased from 225 to 250 °C by sandwiching the GaSb between ultrathin Sb having an own *T_x_* of about 300 °C. Such a heterostructure is attractive because it is based on immiscible stable phases that will again form when cooling from the liquid state. In contrast, when the same GaSb is sandwiched between Sb_2_Te_3_ layers, its *T*_*x*_ reduced to 200 °C. Our work thus provides further evidence that ultrathin Sb films are highly attractive for phase change memory and photonics applications.

## Experimental Section

### Sample Preparation and Thin Film Growth

Sb thin films and heterostructures were deposited using pulsed laser deposition (PLD) on Si/SiO_2_ substrates for ellipsometry characterizations and on continuous carbon and Si_3_N_4_ TEM grids for scanning electron microscopy (SEM) and scanning/transmission electron microscopy (S/TEM) characterizations. A powder sintered Sb target from K-TECH has been used for deposition. PLD deposition parameters have been optimized for best transfer and yield. Our PLD system has a KrF excimer laser with a wavelength of 248 nm. For Sb thin film depositions, a fluence of 1.5 J cm^–2^, a target–substrate distance of 55 mm, and an argon processing pressure of 10^–3^ mbar were used. The amorphous nature of as-deposited Sb thin films was *in situ* confirmed by a reflective high-energy electron diffraction (RHEED) setup. Since our deposited thin films are few nanometers thick, thickness control during deposition was crucial. Therefore, the number of laser pulses used during the deposition for the specified PLD parameters have been calibrated with thickness measurements using spectroscopic ellipsometry fitting and AFM (by scratching the film surface). In addition, for consistency, Sb thin films of varying thickness have been prepared consecutively with the same PLD settings. Details of the data fitting and thickness extraction are provided in the Supporting Information (see SI-1).

### Spectroscopic Ellipsometry Characterization

Dynamic ellipsometry (DE) measurements were conducted to study the phase transformation of amorphous as-deposited Sb thin films to crystalline thin films. All measurements were done with a J. Woollam UV–vis spectroscopic ellipsometer. An HTC-100 heating stage, attached to the variable-angle spectroscopic ellipsometry (VASE) setup, and the TempRampVASE software controller were used for DE measurements. All DE measurements were conducted in air, at a 70° incidence angle and with a 5 °C min^–1^ heating rate. Before and after phase transformation of Sb thin films, spectroscopic ellipsometry (SE) measurements were collected for all thicknesses. Measurement data of ψ and Δ for both amorphous and crystalline samples of Sb thin films were collected in the spectral range of 300–1700 nm. Multiple measurements at variable angles of incidences have been collected to increase the fitting accuracy and reduce the parameter correlation during fitting. Refractive index (*n*) and extinction coefficient (*k*) have been extracted by fitting the measurement data with the Tauc–Lorentz oscillator model using the WVASE fitting software.

### Transmission Electron Microscopy Imaging

A cross-sectional specimen was prepared using an FEI Helios G4 CX focused ion beam (FIB) to investigate the individual layer morphology from an optical heterostructure device. The layer morphology of the cross-sectional specimen and the plan-view TEM of the as-deposited samples were imaged by the JEOL 2010 TEM operating at 200 kV accelerating voltage.

### Reflectance Simulation and Measurement

Reflectance profiles were calculated using a house-built script based on the transfer matrix algorithm.^30^ Multiple annealing steps were performed to crystallize a single layer at a time. Reflectance measurements of the heterostructure were collected after individual layer crystallization using the same ellipsometry setup. All reported reflectance measurements correspond to the p-polarization reflectance at a 15° angle with respect to normal incidence. We choose this angle of incidence since the ellipsometry has a minimum incidence angle of 12°, and the light source will collide with the detector for lower incidence angles.

## Supporting Information Available

Spectroscopic ellipsometry fitting example with detailed explanation and AFM scans of Sb thin film surfaces for roughness and thickness measurements (Figure S1); TEM images of the as-deposited Sb film with the SAED image and large-area view of heterostructure devices (Figure S2); and dynamic ellipsometry results and spectroscopic ellipsometry scans for LAO-capped Sb thin films on thermal oxide substrates (Figure S3) (PDF)
