# Supplementary material for: Thickness-Dependent Crystallization of Ultrathin Antimony Thin Films for Monatomic Multilevel Reflectance and Phase Change Memory Designs
Source: ACS Appl Mater Interfaces. 2022 Mar 10;14(11):13593–600. doi: 10.1021/acsami.1c23974 (PMC8949766; doi:10.1021/acsami.1c23974)
Supplement: Supplementary file 1 — am1c23974_si_001.pdf [file am1c23974_si_001.pdf]

Supporting Information

Thickness-Dependent Crystallization of Ultrathin Antimony Thin Films for Monatomic Multilevel Reflectance and Phase Change Memory Designs

Daniel T. Yimam* and Bart J. Kooi**

Zernike Institute for Advanced Materials, University of Groningen, Nijenborgh 4, 9747 AG Groningen, The Netherlands

*Email: d.t.yimam@rug.nl

**Email: b.j.kooi@rug.nl

SI 1 – Spectroscopic ellipsometry data fitting and AFM thickness extraction.

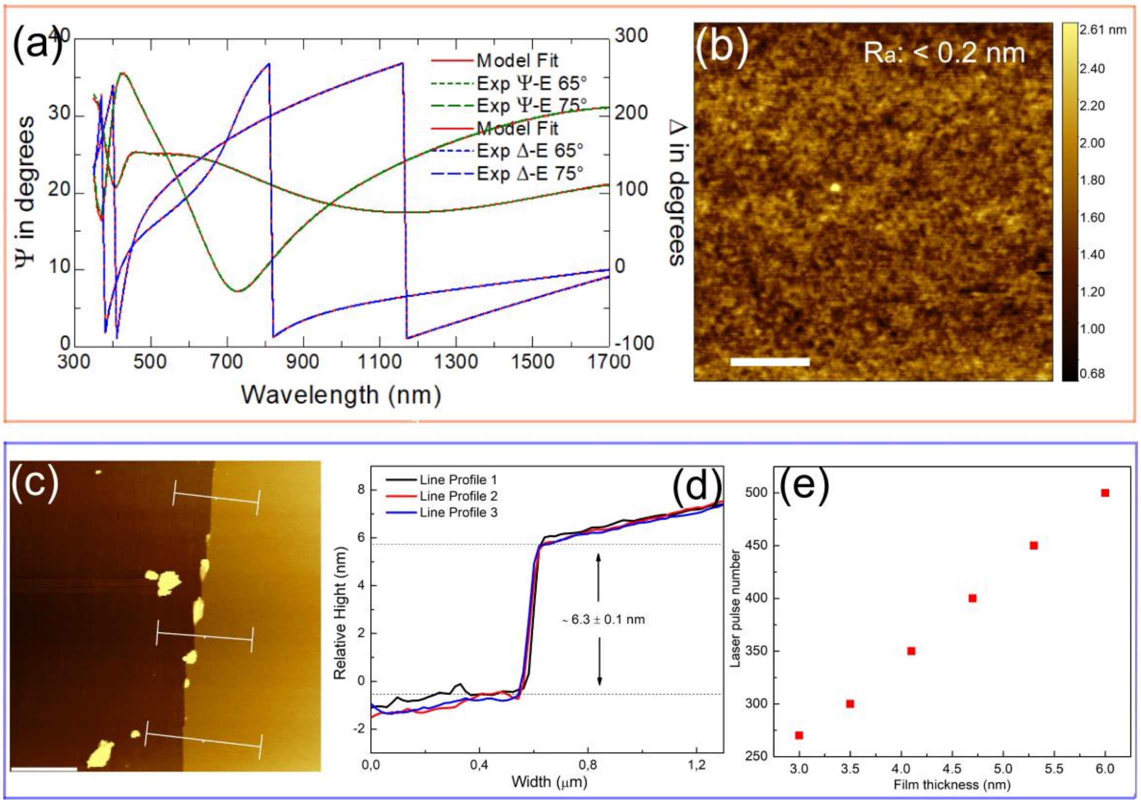

Figure S1. (a) An example of spectroscopic ellipsometry measured parameters of ψ and Δ and the fit generated values from the constructed model for 6 nm Sb thin film on a 300 nm thermal oxide substrate. (b) AFM image of a 400 pulses as-deposited Sb thin film captured in tapping mode showing a very low roughness. The scale bar is 200 nm. (c) Thickness measurement using an AFM scan over a scratched surface. The scale bar is 1 μm. (d) The difference in height from the line profiles indicates an approximate thickness for the thin film of 6.3 nm. (e) Laser pulse number Vs. film thickness relationship showing a linear relation. The pulse number Vs. thickness relationships were derived by combining initial AFM thickness results with spectroscopic fitting for thicker and thinner films.

In spectroscopic ellipsometry, linearly polarized light is reflected by a sample. The change in phase and intensity, before and after reflection, is used to extract information about the sample. The interaction of the initial linearly polarized light with the sample will produce transmission

and reflection light intensities upon reaching multiple interfaces inside the sample. The reflection and transmission of partial intensities on the interfaces can be represented by a collection of equations called the Fresnel equations. The total reflected light intensities for both the perpendicular (s-polarized) and parallel (p-polarized) lights (Rs and Rp) for the specific incidence angle are directly related to the ellipsometry measured parameters (ψ and Δ). The complex reflection coefficient (ρ), which is the ratio of the Rp and Rs, relate the reflectance intensities with the measured parameters as:

$$\rho = \frac{Rp}{Rs} = \tan(\psi) \exp\ (i\Delta)$$

Where ψ and Δ represent the change in intensity and phase of the detected light from the original linearly polarized light.[1,2]

The measurement parameters of ψ and Δ by themselves do not represent any material properties. Therefore, they do not provide enough information about the measured sample (except for bulk samples and dynamic ellipsometry measurement discussed in section SI-3). In turn, they have to be converted into optical material properties like an index of refraction (n) and extinction coefficient (k) and physical parameters like film thickness and roughness. Therefore, a model has to be constructed based on individual layers present in the sample to extract the physical and optical properties of the measured sample. Each layer in the model is represented by dispersion relations containing fitting parameters. For our samples, a commercially available software, WVASE, produced by the J.A. Woollam Company, was used to perform the data fitting.

Measurement data of ψ and Δ for Sb thin films of variable thickness were collected in the 300 – 1700 nm spectrum range. The measurements were done for both as-deposited and crystalline films. Figure S1 (a) shows an example of measured parameters of ψ and Δ collected at 65° and 75° angle of incidence for a 6 nm thick Sb film. In addition, the fitted values of the measurement

are also presented in the figure. For the data fitting, a single Tauc – Lorentz function was used to represent the optical dispersion relation of our Sb thin film. The fitting accuracy is evaluated by the maximum likelihood calculator function in the WVASE software.[3] For all our fittings, small Mean Squared Error (MSE) values were achieved (<10), which indicates a perfect resemblance of our model to the measured values as can also be clearly observed in Fig. S1a.

One fitting parameter was roughness, described by an effective medium approximation (EMA), where the value was assumed by 50% of the topmost layer and 50% void.[2] A roughness layer thickness value of zero produced the best results in our fitting, indicating a smooth surface of our deposited thin films. Next, AFM scans in tapping mode were performed to confirm our deposited thin films' full coverage and smoothness. An example of such a scan is shown in Figure S1 (b) for an Sb thin film of 400 pulses (4.7 nm in thickness). The Gwyddion software package was used to analyze the AFM images.[4] The mean roughness value (Ra) of < 0.2 nm was found in this case, indicating a very smooth surface. The full coverage and smoothness of the 500 pulses Sb film is also visible on the right side of the scratch in Figure S1 (c), where a film with a thickness of only 6.3 nm (on the right) shows uniform contrast compared to the bare surface (on the left).

So, we carefully scratched our film surface and performed an AFM measurement as a second thickness confirmation and an initial thickness input for ellipsometry fitting. Figures S1 (c) and (d) show the AFM scan image, and the average results of the line scan over the scratched area, respectively. We extracted an average of 6.3 nm thickness from the line profiles, which was used as initial input for the ellipsometry data fitting. After this fitting, a thickness of 6.0 nm was extracted. Based on these results for the 500 pulses Sb film, the fitting was also performed for the Sb film produced with fewer pulses and then the input thickness for the fitting was adapted directly proportional to the number of pulses used.

SI 2 – TEM and SAED images

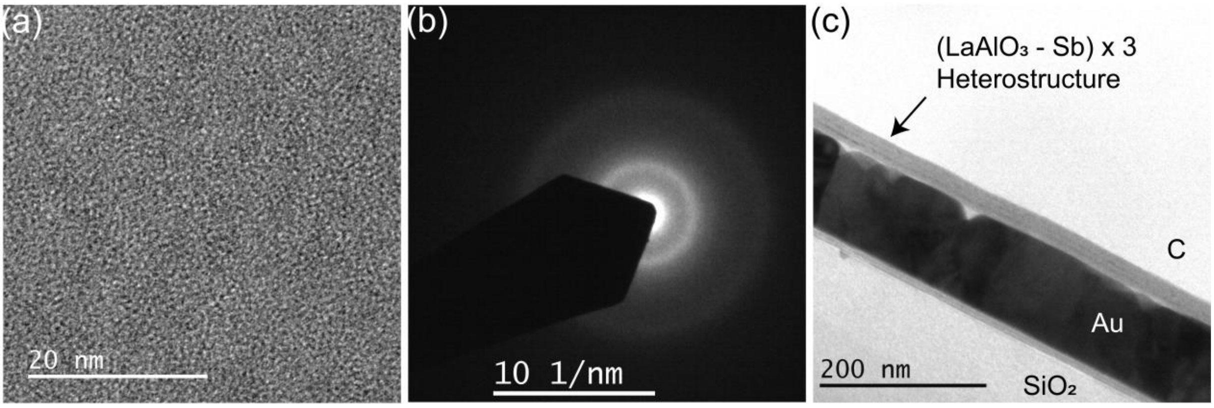

Figure S2. (a) High resolution TEM image of as-deposited 6-nm Sb thin film on SiN grid. (b) Selected area electron diffraction (SAED) image of the as-deposited Sb thin film. (c) A cross-sectional TEM image of the heterostructure device (similar to Fig. 3b of main text) shows a smooth coverage of the Sb and $LaAlO_3$ layers on the gold substrate.

Our PLD has a Reflection High Energy Electron Diffraction (RHEED) setup for in-situ monitoring of the deposited thin films. The setup was initially used to confirm the amorphous nature of the as-deposited Sb thin films inside the chamber. We also prepared Sb thin films using Si_3N_4 TEM grids for TEM analysis. Figure S2 (a) and (b) show examples of TEM analysis results for a 6 nm Sb as-deposited thin film. The plan-view image and the selected area electron diffraction (SAED) provide additional evidence for the amorphous nature of the deposited thin films. An additional TEM image, over a large area, for the heterostructure device based on multiple Sb thin film layers with varying thicknesses is given in Figure S2 (c). Smooth and complete coverage of the heterostructure stacks of Sb and $LaAlO_3$ films is visible.

SI 3 – Dynamic ellipsometry measurements

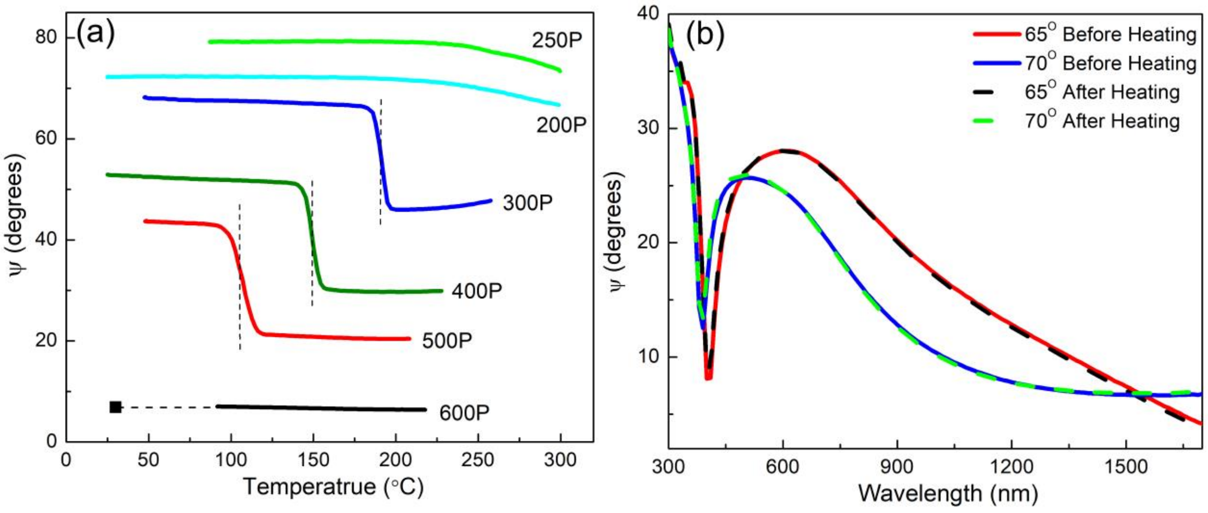

Figure S3. (a) Intensity change due to phase transformation of Sb thin films with variable film thicknesses and $LaAlO_3$ capping. (b) Before and after annealing for 600 pulses Sb thin film, spectroscopic scans show no change in measurement values since phase transformation already occurred at room temperature.

Dynamic ellipsometry (or in-situ measurements) provides insight into material property changes over time. For in-situ monitoring of thin-film growth or studying phase transformation, DE provides non-invasive and easy measurement results relatively quickly. In spectroscopic ellipsometry, measurement data for each wavelength in the spectral range is collected. In DE, specific wavelength values are selected, and measurement parameters are collected over time. The choice of wavelength is based on prior spectroscopic ellipsometry measurements of the amorphous and crystallized films, showing at which specific wavelength a strong contrast in the measured signal can be expected during crystallization.

Multiple measurement parameters can be used to study the phase transformation. This study mainly focused on the ψ parameter and the pseudo index of refraction (<n>) values extracted by assuming the heterostructure as a bulk. Figure S3 (a) show an example of DE measurements

for Sb thin films of variable thicknesses. The films are pulsed laser deposited and are capped by a 4 – 5 nm thick transparent $LaAlO_3$ (LAO) layer. Measurement parameters presented here are collected at a 70-degree angle of incidence and λ = 1500 nm. As shown for 300, 400, and 500 pulses thin films, the measurement parameter ψ show a dramatic drop in value at a specific temperature. As stated in the main text, this temperature corresponds to the sample's crystallization temperature (Tx).

The results presented here for Sb thin films with LAO capping produced similar Tx values as Sb thin films without capping (results presented in the main text). Although for entirely different reasons, thinner (200P and 250P) and thicker (600P) Sb thin films do not show any crystallization-induced DE parameter changes during each measurement. Crystallization does not occur for thinner films because the Tx is high and beyond the measurement range used (maximum is 300 ^{O}C). However, for 600P Sb thin film, the phase transformation already happened at room temperature before the measurement, and annealing to higher temperatures then does not produce any property changes. Figure S3 (b) shows spectroscopic scan measurements of the 600P Sb thin film before and after annealing to 220 ^{O}C. For all incidence angles used, no change in the measured ψ values is observable since no phase transformation occurred.

References

1) M. Losurdo, K. Hingerl, Ellipsometry at the Nanoscale, Springer Science & Business Media, 2013.
2) H. Fujiwara, Spectroscopic ellipsometry: principles and applications, John Wiley & Sons, Chichester, England; 2007.
3) Guide to Using WVASE 32: Spectroscopic Ellipsometry Data Acquisition and Analysis Software, J. A. Woollam Company, Incorporated, 2008.
4) David Nečas, Petr Klapetek, Gwyddion: an open-source software for SPM data analysis, *Cent. Eur. J. Phys.* **10**(1) (2012) 181-188
